# Stimulation of ACE inhibitory and improving α‐amylase and α‐glucosidase and antioxidant activities of semi‐prepared and dry soup by incorporating with date kernel powder

**DOI:** 10.1002/fsn3.3170

**Published:** 2022-12-13

**Authors:** Maryam Mousavi, Vajiheh Fadaei, Behrouz Akbari‐adergani

**Affiliations:** ^1^ Department of Food Science and Technology, Shahr‐e‐Qods Branch Islamic Azad University Tehran Iran; ^2^ Food and Drug Laboratory Research Center, Food and Drug Administration Ministry of Health and Medical Education Tehran Iran

**Keywords:** angiotensin‐converting enzyme, date kernel, dry soup, α‐amylase, α‐glucosidase

## Abstract

Date kernel as a functional food component has a special importance due to its rich nutritional profile, low price, and ease of access. For this, in this research, the sub‐product was used for formulation of semi‐prepared dry soup (SPDS); the effect of adding 0 (S1 = control), 2 (S2), and 4 (S3) %w/w date kernel powder (DKP) on physicochemical, nutritional, and organoleptic properties and beneficial effects of SPDS samples were evaluated. The results revealed that S2 and S3 samples were different from the control sample in some physicochemical properties so that viscosity increased 1.27 and 1.52 times and a* raised 5.6 and 8.5 times, respectively, while L* decreased 0.94 and 0.88 times and b* reduced 0.92 and 0.8 times, respectively. The nutritional properties of S2 and S3 samples compared with the control sample improved. Also, differences were observed in the beneficial effects of S2 and S3 compared with the control sample as total polyphenol content (TPC) increased 1.06 and 1.11 times, respectively (*p* < .05); antioxidant activities (AA) of S2 and S3 samples were 8.04 and 6.01 mg/ml and angiotensin‐converting enzyme (ACE) inhibitory activities were measured to be 8.2 and 7.86 mg/ml, respectively; also, α‐amylase and α‐glucosidase inhibitory activities of S2 and S3 samples were observed 4.48% and 5.70%, and 4.59% and 6.36%, respectively. From the organoleptic aspect, S3 had the highest acceptability. Generally, it is concluded that with the addition of DKP (maximally 4%w/w) to SPDS formulation, a functional soup could be produced considering the rich nutritional profile of DKP.

## Graphical abstract

Date kernel powder (DKP) could be considered as the potential functional ingredient in healthy food products. DKP improved α‐amylase and α‐glucosidase and antioxidant activities of semi‐prepared and dry soup (SPDS). SPDS with improved nutritional properties was provided using DKP incorporation.

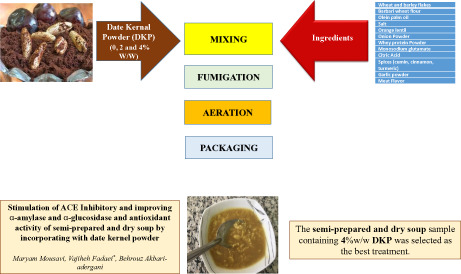

## 1 INTRODUCTION

Semi‐prepared dry soup (SPDS) is produced in a dried form after processing and packaged in a suitable way; it is consumable after the addition of water or other suitable liquids as well as desirable food components and boiling. SPDSs have received a lot of attention in recent years because of their favorable shelf life, easy transportation, and short time required for their preparation (Niththiya et al., 2014). Now, if functional ingredients are employed in preparation of this type of soups, their value will be doubled. Since eating soup is the easiest way for receiving nutrients and with increasing workload and not having enough time for most people in the community to prepare this type of product in the food industry, ready‐to‐eat soups are produced in powder form, it is expected that by using a functional component as a value‐added food ingredient, soups with high nutritional and healthful values are prepared and people can receive a major portion of nutrients and healthful components through the consumption of soup.

In recent years, with the introduction of functional foods and increasing consumer awareness of the relationship between health and food consumption, the demand for foods with significant health‐giving effects has increased. Currently, food factories are trying to generate a product with numerous health‐giving effects. The health‐giving effects of functional foods are attributed to their healthful bioactive compounds, and date kernel, due to its nutritional and health‐giving properties mentioned below, is among the compounds that can be used as a functional food ingredient.

Depending on the date variety, date kernel comprises 10% (on average) of the total weight of the date and it is produced in large quantities as waste in date processing factories. Date kernel is odorless and has a light to dark brown color and a slightly mild bitter taste (Hamada et al., 2002). Phenolic acids, flavonoids (epicatechin, catechins, procyanidins, quercetin, naringinin, rutin, luteolin, chrysoerio, tricin, and apigenin), anthocyanins, carotenoids (β‐carotene, lutein), tocopherols (α and γ‐tocopherol, tocotrienol), phytosterols (β‐sitosterols), fatty acids (oleic, linoleic, palmitic, and lauric), and carboxylic acids have been reported among bioactive compounds of this sub‐product (Alharbi et al., 2021). Date kernel contains 7.1%–10.3% moisture, 46%–51% acid detergent fiber, 65%–69% neutral detergent fiber, 5%–6.3% protein, 9.9%–13.5% fat, and 1%–1.8% ash (Hamada et al., 2002). TPC of date kernel is 48.64 mg gallic acid/100 g date. The dietary fiber of date kernel is 58% from which 53% is soluble dietary fiber. Semi‐processed date kernel has equal protein to wheat flour (Wahini, 2016). Date kernel protein plays an important biological role in our body due to its essential amino acids (Bouaziz, 2020). Different insoluble fibers including cellulose and hemi‐cellulose are major carbohydrates of date kernel (Ghnimi et al., 2017). From ancient time, in the Iran's regions under date cultivation, date kernel powder (DKP) has been traditionally used to treat some diseases such as hypertension, headache, etc. Date kernel has unique properties such as antioxidant, antimicrobial, antiviral, hypoglycemic, and anti‐cancer characteristics (Al‐Qarawi et al., 2005).

Considering the available literature, most of the studies are devoted to the effect of DKP on bread quality and bakery products and except in one case (Ambigaipalan & Shahidi, 2015), functional properties of prepared food products have not been discussed (Almana & Mahmoud, 1994; Bouaziz et al., 2010, 2020; Ghasemi et al., 2020; Najjar et al., 2022; Platat et al., 2014, 2015; Vinita, 2016). Of course, some researchers evaluated physicochemical and organoleptic properties of non‐bakery products rich in DKP or date kernel fiber (Amany et al., 2011; Asghari‐pour et al., 2018; Bouaziz, 2020; El Sheikh et al., 2014; Fikry et al., 2019; Wedad & El‐Khol, 2018). It should be noted that none of these studies have mentioned the adverse toxicological effects of date kernel powder; however, El Sheikh et al. (2014) have confirmed in vitro cytotoxic activity of roasted date kernels. Also, considering the available literature, no research on the evaluation of the effect of this valuable and cheap functional sub‐product on the quality of dry soup has still been undertaken (Abdel‐Haleem & Omran, 2014; Niththiya et al., 2014; Olubi et al., 2021; Upadhyay et al., 2017). On the other hand, based on the studies, the highest content of antioxidant compounds in dates is included in their kernels, which encompass phenolic compounds (Wahini, 2016). Also, date kernel has therapeutic effects and high level of dietary fibers which can be a good source for investigating α‐amylase and α‐glucosidase inhibitory activities. Nevertheless, a large amount of this functional sub‐product is wasted in factories producing date by‐products. For this reason, in this research, DKP is used as a health‐giving food component in catering functional SPDS. On this basis, the goal of this research was to formulate SPDS based on cereals and DKP and evaluation of its properties from different aspects.

## 2 MATERIALS AND METHODS

### 2.1 Materials

Olein palm oil from Margarin Public Company, wheat and barley flakes from Abshar Sabz, Barbari wheat flour from Setareh Mortazavi Company, refined iodine‐free salt from Pars Namak Kaveh Company, orange lentil from Golestan Company, date (*Phoenix dactylifera L*.) kernel powder (variety Mazafati) from Pilar Company, onion powder from Rashak Company, whey protein powder from Shirpooyan Company, citric acid from Ensign Company, garlic powder from Rashak Company, Meat Flavor from Symrise Company, monosodium glutamate from Foodchem and spices from Behsab Company were prepared. All chemicals required for carrying out tests were supplied by Merck Company except sucrose, angiotensin‐converting enzyme (ACE), diphenyl 2‐picrylhydrazyl, hypuryl‐His‐Leu, α‐amylase and α‐glucosidase purchased from Sigma Company.

### 2.2 Preparation of semi‐prepared dry soup samples

All required materials were approved by the quality control unit were weighed individually (according to Table 1) for the preparation of SPDS samples. Subsequently, all weighed materials were mixed in a special mixer and transferred to a particular silo for packaging in 75 g laminated bags (three layers of low‐density polyethylene foil/aluminum/low‐density polyethylene). Random sampling was carried out from the produced soups, and the relevant tests were performed 7 days after production.

**TABLE 1:** Introduction of treatments used in research

Integration	Treatment		
	S_1_ (%)	S_2_ (%)	S_3_ (%)
DKP	0	2	4
Wheat and barley flakes	64.2	62.2	60.2
Barbari wheat flour	12	12	12
Olein palm oil	8	8	8
Salt	6	6	6
Orange lentil	2.7	2.7	2.7
Onion powder	2	2	2
Whey protein powder	1.6	1.6	1.6
Monosodium glutamate	1	1	1
Citric acid	0.6	0.6	0.6
Spices (cumin, cinnamon, turmeric)	1.1	1.1	1.1
Garlic powder	0.7	0.7	0.7
Meat flavor	0.1	0.1	0.1
Total	100	100	100

It should be mentioned that since the color of DKP had a great effect on the color of soups, first, the formulation was designed to prepare a control soup sample so that its color would not be substantially affected by the addition of low percentages of DKP. After the approval of organoleptic properties of control soup sample by 10 trained panelists, the next stage of research was started for the determination of experimental treatments. Indeed, 15 treatments having 0%–7% DKP (0%, 0.5%, 1%, 1.5%, 2%, 2.5%, 3%, 3.5%, 4%, 4.5%, 5%, 5.5%, 6%, 6.5%, and 7%) were prepared as soup samples enriched with higher than 4% DKPs had an astringent taste and a dark color; thus, they were unacceptable by panelists and removed, and samples were selected according to Table 1.

### 2.3 Experiments on date kernel powder and soup samples

For preparation of soups, 75 g SPDS was mixed with 1 L cold water and boiled for 20 min at mild temperature. Then, the prepared and cooled soup was poured in a mixer, and the mixed sample (with ambient temperature) was tested.

#### 2.3.1 Physicochemical properties

Titratable acidity was calculated by titrating the samples with 0.1 N NaOH (Niththiya et al., 2014) and pH was measured by a pH meter (Schott GERATE; Fikry et al., 2019).

Moisture content was evaluated using dry air oven (Fikry et al., 2019).

Color parameters were measured with a Chroma‐meter (TES 135A, Taiwan) and reported in terms of L*, a*, and b* values (Fikry et al., 2019).

The viscosity of soup samples was measured by a rotational Brookfield viscometer (RVDV‐ II+ pro). After initial tests, DV64 was selected as the suitable spindle and viscosity was reported at room temperature in terms of Pa/s after 30 s (Abdel‐Haleem & Omran, 2014).

For calculation of rehydration, 2 g of functional semi‐prepared dry soup was mixed with 20 ml distilled water at a fixed speed (100 rpm) and rehydrated in a water bath at a given temperature (45°C) for 10 min; then, samples were filtered, weighed and the relevant factor was calculated (Abdel‐Haleem & Omran, 2014).

#### 2.3.2 Nutritional properties

Fat content was determined using Soxhlet extraction **(**El Sheikh et al., 2014), protein content by Kjeldahl method (Bouhlali et al., 2015), carbohydrate content by titration (McCleary et al., 2019), minerals (iron, zinc, sodium, potassium, calcium, magnesium, copper, and selenium) by Bouhlali et al. (2015) method, ash content by direct ignition (El Sheikh et al., 2014), and soluble, insoluble as well as total fibers by Abdel‐Haleem and Omran (2014) method were measured.

Total calories were estimated by Equation (1) (Bouhlali et al., 2015):

(1) $$\begin{array}{c} \text{Total energy}\;\left(\text{calories}\right)=\left(\text{Total fats}\;\mathrm{per}\;100\;g\right)\times 9\\ +\left(\text{Total carbohydrates}\;\mathrm{per}\;100\;g\right)\times 4+\left(\text{Total proteins}\;\mathrm{per}\;100\;g\right)\times 4. \end{array}$$

#### 2.3.3 Beneficial effects

The total polyphenol content (TPC) was calculated through the Folin–Ciocalteu method and reported in terms of mg gallic acid per 100 g of sample (Fikry et al., 2019). According to the Folin–Ciocalteu procedure, the sample and Folin–Ciocalteu reagent were thoroughly mixed in a volumetric flask. After 3 min, 5 ml of 10% Na2CO3 solution was added, and the mixture was left for 1 h. The absorbance of the mixture was determined at 760 nm by using a spectrophotometer (UV2100). The total concentration of phenolic compounds was determined by comparison with the absorbance of chlorogenic acid as standard.

Antioxidant activity (AA) was measured through the evaluation of free radical scavenging capacity of 2,2‐diphenyl−1‐picrylhydrazyl (DPPH) and reported in terms of IC_50_ (Bouhlali et al., 2015). IC_50_ is inhibitory concentration (mg/ml) required for the reduction of DPPH radicals to 50% of its original content and able to inhibit 50% of its activity. The lower IC_50_, the higher antioxidant capacity. The sample was mixed with 0.4 mmol/LDPPH radical in ethanol. The mixture was vigorously shaken and left for 10 min. The absorbance of the mixture was determined at 525 nm with a spectrophotometer (UV2100). The radical scavenging activity was calculated using Equation (2) as follows:

(2) $$\text{Inhibition}\;\left(\%\right)=\left[1-\left({\mathrm{abs}}_{\text{sample}}/{\mathrm{abs}}_{\text{control}}\right)\right]\times 100.$$

To measure ACE inhibition, first, 75 g of sachet content was mixed in individual containers with 1 L cold water and boiled gradually by thermal energy for 20 min while stirring; after cooling down to room temperature, it was filtered through Whatman filter paper and the filtered solution was assayed through Balthazar et al. (2019) method. In brief, to determine the amount of ACE inhibitory activity, 80 μl of the filtered solution was mixed with 200 μl of 5 mM hyporyl L‐histidyl L‐leucine (HHL) solution and incubated for 3 min at 37°C. Samples and HHL were prepared in 100 mM borate buffer (pH = 3.8) containing 300 mM sodium chloride. Next, 20 μl of 0.1 U/ml solution of ACE was added. Then, they were incubated for 30 min; and after that, the enzymatic reaction was stopped by adding 250 μl of 0.05 M HCl solution. The freed hippuric acid was extracted with about 2 ml of ethyl acetate and evaporated at a temperature of about 90°C for about 10 min. The residue was dissolved in 1 ml of distilled water and its absorbance was read at 228 nm by an ELISA reader. ACE inhibitory activity reported as IC_50_ was calculated according to Equation (3). IC_50_ is inhibitory concentration for inhibition of 50% of ACE activity in terms of mg/ml. Lower IC_50_ values show higher inhibition percentages.

(3) $$\mathrm{ACE}\;I.\left(\%\right)=\frac{\;\left(B-A\right)}{\left(B-C\right)}\times 100.$$

*A*: hippuric acid absorption in the presence of ACE inhibitor. *B*: absorption in the absence of ACE inhibitor. *C*: absorption in the absence of ACE.

α‐amylase and α‐glucosidase inhibitory activities were measured through Laoufi et al. (2017) method. The reaction mixture for determining the α‐amylase inhibitory activity contains the following: 200 μl of the filtered solution, 200 μl of 0.02 M sodium phosphate buffer (pH 6.9; 6.7 mM NaCl) containing 1.3 U/ml of porcine pancreatic α‐ amylase solution (PPA). The reaction medium was pre‐incubated at 37°C for 5 min, and then 200 μl of 0.4% starch solution in the above buffer was added and incubated at 37°C for 10 min. Six hundred microlitersl of DNSA solution was added to the reaction and placed in a boiling water bath for 7 min, then cooled down in cold water. The reaction mixture was then diluted after adding 1 ml of distilled water and the absorbance was measured at 540 nm. To eliminate the absorbance produced by filtered solution, appropriate solution controls with the filtered solution and except the enzyme were also included. Commercial inhibitor acarbose was used as a positive control at a concentration range of 0.040–2.670 mg/ml. As a blank buffer solution was used instead of substrate. The tube with enzyme solution but without the filtered solution/acarbose served as the control with total enzyme activity. For determining the α‐glucosidase inhibitory activity, the filtered solution (500 μl) and 1000 μl of 0.1 M potassium phosphate buffer (pH 6.90) containing α‐glucosidase solution (1.0 U/ml) were incubated in water bath (25°C) for 10 min. *p*‐Nitrophenyl‐α‐d‐glucopyranoside solution (5 mM, 500 μl) in 0.1 M potassium phosphate buffer (pH 6.90) was then added to each tube and the mixtures were re‐incubated at 25°C for 5 min. Absorbance readings were recorded at 405 nm and the control (buffer in place of the filtered solution) was recorded as well.

The enzyme inhibition rate expressed as a percentage of inhibition was calculated using Equation (4).

(4) $$\text{Inhibition of enzyme activity}\;\left(\%\right)=\left(\left(C-S\right)/C\right)\times 100.$$

*C*: absorbance of the control (100% enzyme activity). *S*: absorbance of the tested sample.

#### 2.3.4 Organoleptic properties

Organoleptic properties of DKP were measured through evaluation of its color, flavor, taste, and odor.

Organoleptic properties such as flavor, odor, oral and non‐oral texture (mouthful, pouring, stirring, and spoon able), color, and overall acceptability of soup samples were measured by a 5‐point hedonic system. Evaluation levels included (from 1 to 5): 1 = inconsumable or very weak, 2 = unacceptable or weak, 3 = acceptable or average, 4 = satisfactory or good, 5 = very satisfactory or very good (Abdel‐Haleem & Omran, 2014).

Sensory evaluation was carried out by 100 untrained panelists (30 men and 70 women in the range of 25–60 years).

### 2.4 Statistical analysis

A completely randomized design was used to analyze and evaluate the data obtained from the different experiments. Analysis of variance and comparison of means (three replicates for each experiment) were carried out through SPSS software version 16.0 (Duncan's multiple range test and confidence level of 95%).

## 3 RESULTS AND DISCUSSION

### 3.1 Physicochemical properties

#### 3.1.1 Titratable acidity and pH

Table 3 shows pH and acidity changes of soup samples. With the increase in DKP percentage in functional soup samples, pH decreased and acidity increased (*p* < .05), and S1 and S3 had the highest pH value and acidity, respectively. Reduced pH and elevated pH could be attributed to the low pH and high acidity of DKP (Table 2). The reason for high acidity of DKP is the presence of acidic compounds such as caffeic acid, gallic acid, p‐coumaric acid, ferulic acid, and vanillic acid in it (Al Harthi et al., 2015; Al‐Farsi & Lee, 2011).

**TABLE 2:** Characterization of date kernel powder^a^

Characteristics	Treatment
pH	5.63
Titratable acidity (g/100 g)	0.003
Moisture (g/100 g)	2.93
*L* ^a^	16.91
*a* ^a^	14.28
*b* ^a^	7.18
Fat (g/100 g)	15.07
Protein (g/100 g)	6.98
Carbohydrate (g/100 g)	77.03
Ash (g/100 g)	0.94
IDF (g/100 g)	23.15
SDF (g/100 g)	34.18
TDF (g/100 g)	57.20
K (ppm)	1857
Cu (ppm)	6455.8
Ca (ppm)	1187
Mg (ppm)	1244
Na (ppm)	146.7
Fe (ppm)	2492
Zn (ppm)	265.83
Se (ppm)	2682
Calori (kcal/kg)	4237
AA (mg/ml)	0.074
TPC (mg GAE/g)	1.67
ACE inhibitory activity (mg/ml)	9.1
α‐glucosidase Inhibition (%)	19.9
α‐amylase Inhibition (%)	34.57
Sensory properties	Good

^a^ Numbers are the average of three repetitions.

#### 3.1.2 Moisture, viscosity, and rehydration

Table 3 shows the changes in moisture content, viscosity, and rehydration of soup samples. Increasing the percentage of DKP in soup samples decreased moisture content and rehydration (*p* > .05) and promoted viscosity (*p* < .05); viscosity of S2 and S3 was 1.27 and 1.52 times higher than control sample, and, in fact, S3 had the highest viscosity.

**TABLE 3:** Physicochemical properties of semi‐prepared dry soup samples* (mean ± standard deviation)

Treatment characteristics	S1 (DKP = 0)	S2 (DKP = 2)	S3 (DKP = 4)
pH	4.6^a^ ± 0.0	4.6^a^ ± 0.01	4.45^b^ ± 0.05
Acidity (g/100 g)	0.0076^b^ ± 0.00037	0.0074^b^ ± 0.00000	0.0082^a^ ± 0.00037
Moisture (g/100 g)	5.14^a^ ± 0.053	4.98^a^ ± 0.624	4.80^a^ ± 0.264
*L* *	61.9^a^ ± 0.1	57.9^b^ ± 0.4	54.4^c^ ± 0.8
*a* *	−1.47^c^ ± 0.115	3.15^b^ ± 0.359	6.03^a^ ± 0.302
*b* *	17.86^a^ ± 0.066	16.37^b^ ± 0.865	14.30^c^ ± 0.36
Viscosity (Cp)	969^c^ ± 51	1221^b^ ± 87	1472^a^ ± 127
Rehydration (g/100 g)	874^a^ ± 108	599^ab^ ± 154	542^b^ ± 172

Abbreviation: DKP, Date Kernel Powder (%w/w).

* Means with different subscripts differ significantly (*p* < .05).

Reduced moisture content is ascribed to the availability of insoluble cellulose fibers, hemi‐cellulose, and lignin in DKP, which, owing to the lack of ability to absorb and hold water, caused the decrease in moisture content of DKP (Al‐Farsi & Lee, 2008). Also, hydration (swelling and water retention capacity) characteristics of date kernel dietary fiber have been reported to be lower than fibrex (Shokrollahi & Taghizadeh, 2016). The dwindled moisture content of soup samples enriched with DKP is due to the replacement of DKP with wheat (7% moisture) and barley (5% moisture) as these ingredients possess higher moisture content than DKP (3% moisture).

The viscosity of final product is dependent on the type of raw/initial material. Abdel‐Haleem and Omran (2014) introduced viscosity as the indicator of concentration in dried vegetable soup fortified with legumes so the addition of thickening components to the soup formulation affects the viscosity of the product; also, they reported that when dry foods are recombined, they should exhibit acceptable textural, visual, and sensory properties with a minimum of hydration time. Niththiya et al. (2014) reported increased viscosity after formulating instant soup powder with Borassus flabellifer tuber flour and local vegetables. Compounds with the capacity of moisture absorption result in the increase in viscosity and stability of product (Elleuch et al., 2011; Figuerola et al., 2005). The ability to absorb water in DKP might be attributed to soluble dietary fiber and protein. Water‐soluble polysaccharides of date kernel also own the ability to absorb water and increase viscosity. Because of hydroxyl groups and ability to interact with water molecules through hydrogen bonds and, as a result, increased ability of water holding, the fiber of DKP causes increased viscosity and moisture of enriched product (Bouaziz et al., 2010, 2020; Najjar et al., 2022).

The results of researches related to the effect of moisture are different based on the formulation of products enriched with DKP. Ambigaipalan and Shahidi (2015) reported that the increase in DKP leads to the increase in moisture content in muffin samples. Ghasemi et al. (2020) and Platat et al. (2015) expressed no significant change in moisture content of sponge cake and pita bread after elevating the percentage of DKP. However, Asghari‐pour et al. (2018) and Vinita (2016) stated the reduced moisture content of puff and biscuit samples, respectively, after the addition of DKP. Also, Shokrollahi and Taghizadeh (2016) reported an increase in firmness of bread fortified with higher than 2.5% of DKP.

#### 3.1.3 Color

Table 3 displays the changes in color indices of soup samples. L*, a*, and b* are representatives of brightness, redness‐greenness, and blueness‐yellowness, respectively. Intensification of the percentage of DKP in soup samples decreased L* and b* values but increased a* (*p* < .05). Brightness index of S2 and S3 was 0.94 and 0.88 lower compared with the control sample, while their a* was 5.6 and 8.5 times higher and b* was 0.92 and 0.80 lower than those of the control sample.

Natural colorants of date kernel such as flavonoids, anthocyanins, and carotenoids affect its color (Alharbi et al., 2021). These colorants are polyphenols of DKP (Bouaziz et al., 2020). The intensity of the soup color increased with increasing the level of replacement of barley and wheat flakes with DKP, which indicates a significant direct relationship between the amount of DKP and darkness of soup color. Also, the darkness of the product can be related to the presence of short‐chain carbohydrates and proteins in DKP, which intensify the Millard reaction in the product, and, as a result, decreases the brightness of product (Bouaziz et al., 2010, 2020). The color of DKP influences a* value too. The reduction of b* value reveals yellowness of soup samples. It should be noted that the color of DKP is very different from the color of wheat and barley flakes, and, as a result, impacts the darkness of the product.

Platat et al. (2014) stated that the color of date kernel could be representative of its antioxidant and nutritional properties so that the more intense the greenness‐redness is, the higher the phenolic compounds and total antioxidant capacity will be and the more intense the blueness‐yellowness is, the higher the phenolic compounds, flavonoids, catechins, total antioxidant capacity and the lower the dietary fiber will be. Bouaziz (2020) reported similar results about color values after the addition of insoluble fibers of date kernel as insoluble fiber concentrate to turkey burgers. Also, Shokrollahi and Taghizadeh (2016) and Bouaziz et al. (2010, 2020) confirmed the decrease in brightness of bread enriched with fiber and DKP. Najjar et al. (2022) attributed the darkness of cookies to the darkness of added date kernel.

### 3.2 Nutritional properties

#### 3.2.1 Fat, protein, carbohydrate, and ash

Table 4 displays the changes in fat, protein, carbohydrate, and ash content of soup samples. Increasing the percentage of DKP resulted in the increase in fat content of functional soup samples (*p* < .05), partial decrease in protein content and no significant change in carbohydrate and ash contents.

**TABLE 4:** Nutritional properties of semi‐prepared dry soup samples* (mean ± standard deviation)

Treatment characteristics	S1 (DKP = 0)	S2 (DKP = 2)	S3 (DKP = 4)
Fat (g/100 g)	13.49^b^ ± 0.04	13.57^a^ ± 0.04	13.61^a^ ± 0.04
Protein (g/100 g)	11.03^a^ ± 0.11	10.96^a^ ± 0.04	10.91^a^ ± 0.04
Carbohydrate (g/100 g)	70.4^a^ ± 0.11	70.4^a^ ± 0.03	70.4^a^ ± 0.10
Ash (g/100 g)	5.052^a^ ± 0.03	5.053^a^ ± 0.02	5.052^a^ ± 0.02
IDF (g/100 g)	11.6^c^ ± 0.15	12.1^b^ ± 0.06	12.7^a^ ± 0.11
SDF (g/100 g)	8.6^c^ ± 0.17	8.9^b^ ± 0.08	9.3^a^ ± 0.02
TDF (g/100 g)	20.2^c^ ± 0.28	20.9^b^ ± 0.08	22.0^a^ ± 0.12
K (ppm)	11381^a^ ± 51	11083^a^ ± 51	11083^a^ ± 51
Na (ppm)	26958^a^ ± 15	26596^ab^ ± 15	26234^b^ ± 27
Ca (ppm)	800^b^ ± 0.00	813^ab^ ± 11	827^a^ ± 11
Mg (ppm)	976^b^ ± 12.2	996^ab^ ± 7.0	1013^a^ ± 21.1
Fe (ppm)	1100^c^ ± 25	1158^b^ ± 14	1217^a^ ± 14
Zn (ppm)	20.8^c^ ± 2.9	30.8^b^ ± 1.4	48.3^a^ ± 3.8
Cu (ppm)	63.5^c^ ± 0.00	201.1^b^ ± 18.3	328.1^a^ ± 18.3
Se (ppm)	417.6^c^ ± 11.3	482.7^b^ ± 22.6	534.8^a^ ± 11.3
Calori (KCal/Kg)	4250 ± 1	4240 ± 1	4230 ± 1

Abbreviation: DKP, Date Kernel Powder (%w/w).

* Means with different subscripts differ significantly (*p* < .05).

It is worth noting that date kernel oil has high antioxidant activity as well as considerable phenolic compounds (Besbes et al., 2005). Tafti et al. (2017) reported that amount of extracted oil date kernel is quite low. The slight decrease in the protein content of soup samples containing DKP compared with the control soup is due to the low protein content of DKP. Considering the replacement of wheat and barley flakes with DKP in the formulation of soup samples, reduction of the protein amount is pretty reasonable. Most of date kernel proteins are insoluble (Hamada et al., 2002). The lack of change in ash and carbohydrate contents of enriched soup samples is due to the similarity of ash and carbohydrate content of DKP to wheat flour ash (Hamada et al., 2002). Generally, the ash content of date kernel is low.

Almana and Mahmoud (1994) reported slight decrease in protein content of Mafrood flat bread containing DKP compared with the control sample. Ambigaipalan and Shahidi (2015) did not observe any change in fat content of muffin samples after the addition of DKP but reported a significant ash increase compared with the control sample. Ghasemi et al. (2020) expressed an increase in protein content and no change in acid‐insoluble ash of sponge cake samples after intensifying the percentage of DKP. The results of the research of Asghari‐pour et al. (2018) also showed the low ash content of puff samples enriched with DKP. Vinita (2016) did not notice any significant change in protein, fat, and ash contents of biscuit samples enriched with DKP compared with the control sample. Platat et al., 2015 did not observe any significant change in the protein content of pita bread enriched with DKP but its fat content increased.

#### 3.2.2 Soluble, insoluble, and total fibers

Table 4 shows the changes in different types of fibers of soup samples. With the increase in the percentage of DKP, different types of fibers raised in soup samples (*p* < .05). The comparison of different types of fibers of enriched soup samples (S2 and S3) with the control soup shows that their SDFs are 1.04 and 1.1 times, IDFs are 1.03 and 1.05 times, and TDFs are 1.03 and 1.09 times higher, respectively; S3 had the highest amount of different fiber types. The higher fiber content of DKP compared with wheat and barley is the reason for the observed results. The high fiber content of DKP provides the possibility of its application as a rich source of fibers in different food formulations (Al‐Farsi & Lee, 2008, 2011; Baliga et al., 2011; Bouaziz et al., 2010; Elleuch et al., 2011; Habib & Ibrahim, 2009; Hamada et al., 2002).

In line with our results, the higher fiber content of foods enriched with date kernel powder has been reported (Almana & Mahmoud, 1994; Ambigaipalan & Shahidi, 2015; Asghari‐pour et al., 2018; Ghasemi et al., 2020; Platat et al., 2015; Vinita, 2016).

#### 3.2.3 Minerals

Table 4 shows the changes in mineral contents of soup samples. With an increase in the percentage of DKP, calcium, iron, zinc, copper, and selenium content of functional soup samples went up (*p* < .05). The comparison of these minerals in enriched soup samples (S2 and S3) with the control soup sample shows their calcium content was 1.01 and 1.03 times, the iron content was 1.05 and 1.11 times, the zinc content was 3.2 and 5.2 times and selenium content was 1.16 and 1.28 times higher than those of the control sample, respectively; S3 allocated the highest minerals contents to itself. The sodium content of soup samples dwindled as a consequence of the increase in percentage of DKP (*p* < .05), while no significant change was observed in potassium and magnesium content (*p* > .05).

The reason for the increase in calcium, magnesium, iron, zinc, copper, and selenium content in enriched soup samples is the presence of DKP; as it is observed in the compounds of DKP (Table 2), DKP is rich in these minerals, and the availability of these minerals is in this order: copper > selenium > iron > potassium > magnesium > calcium > zinc > sodium. Since the iron level is high in date kernel, the importance of this sub‐product in the enrichment of food products feels great. The reason for the low sodium content of enriched soup samples is the reduced level of this mineral in DKP. It should be noted that selenium owns antioxidant capacity and is regarded as an anticancer ingredient; the amount of which depends on the cultivation environment of date (Hamada et al., 2002).

Martinez‐Ballesta et al. (2010) reported that among minerals of date kernel, potassium, and iron are available in higher amounts. Ghasemi et al. (2020) observed an increase in sodium, zinc, calcium, iron, and potassium content of the sponge cake sample with the increase in the percentage of DKP.

#### 3.2.4 Calorie

Table 4 displays calorie changes in soup samples. The increase in the percentage of DKP descended the calorie amount of soup samples (*p* > .05). Fiber‐rich cereals such as wheat and barley cause satiation. In this research, with the replacement of DKP, the amount of barley and wheat flakes was reduced compared with the control sample due to the uniformity of the raw material weight in the formulation of the samples. Despite this, the reduction in calorie was observed; thus, the reason for the slight reduction in calorie of enriched soup samples could be attributed to DKP. Al Juhaimi et al., 2012 reported the energy of kernel powder of some date varieties to be 4340–4795 kcal/kg which is similar to that of DKP used in this study (4237 kcal/kg).

### 3.3 Beneficial effects

#### 3.3.1 Antioxidant activity and total polyphenol content

The changes in AA and TPC of soup samples are inserted in Table 5. AA and TPC of functional soup samples increased through increasing the percentage of DKP (*p* < .05). By comparing TPC of enriched soup samples (S2 and S3) with the control soup sample, it is observed that their TPC was 1.06 and 1.11 times higher, respectively, and S3 had the highest TPC. AA (IC_50_) of S2 and S3 was measured to be 8.04 and 6.01 mg/ml, respectively.

**TABLE 5:** Functional properties of semi‐prepared dry soup samples* (mean ± standard deviation)

Characteristics treatment	AA (mg/ml) (IC_50_)	TPC (mg GAE/g)	ACE inhibitory activity (mg/ml)	α‐Glucosidase inhibition (%)	α‐Amylase inhibition (%)
S1 (DKP = 0)	13.08^a^ ± 0.00	0.456^c^ ± 0.00	8.43^a^ ± 0.02	1.06^b^ ± 0.00	2.49^c^ ± 0.21
S2 (DKP = 2)	8.04^b^ ± 0.34	0.482^b^ ± 0.00	8.20^b^ ± 0.02	4.59^a^ ± 0.00	4.48^b^ ± 0.27
S3 (DKP = 4)	6.01^c^ ± 0.09	0.507^a^ ± 0.00	7.86^c^ ± 0.02	6.36^a^ ± 1.77	5.70^a^ ± 0.13

Abbreviation: DKP, Date Kernel Powder (%w/w).

* Means with different subscripts differ significantly (*p* < .05).

One of the reasons for the application of DKP as a functional food ingredient is its great phenolic compounds (Al‐Farsi & Lee, 2008); the AA of DKP has been reported to be quite high due to its high phenolic content (Ghnimi et al., 2017; Shams Ardekani et al., 2010). In fact, TPC is the most effective factor in AA. Antioxidant compounds with two or more electron donor groups have higher AA (Simić et al., 2007). There is a strong correlation between AA and TPC as well as flavonoids (Bouhlali et al., 2015). Flavonoids of date kernel include luteolin, quercetin, kaempferol, apigenin, and isorhamnetin (Hinkaew et al., 2021). The presence of carotenoids with AA in date kernel has been reported (Alharbi et al., 2021). It should be mentioned that TPC and AA capacity of date kernel are higher than date paste, and is comparable to that of tea and grape seed (Platat et al., 2014). Some compounds such as gallic acid, vanillic acid, ferulic acid, caffeic acid, *p*‐coumaric acid, syringic acid (Amany et al., 2011; Hinkaew et al., 2021), protocatechuic acid and p‐hydroxybenzoic acid (Amany et al., 2011) have been found in the kernel of date varieties. Radfar et al. (2019) detected and measured seven phenolic compounds including chlorogenic acid, caffeic acid, vanillic acid, gallic acid, cinnamic acid, 3,5‐DHB, and 2,5‐DHB in four varieties of date kernel while they reported cinnamic acid as the most frequent phenolic compound in date kernel extract. Besides, polysaccharides (Fan et al., 2010; Jiao et al., 2011; Luo et al., 2010) and plant fibers (Hamada et al., 2002) have AAs as well.

Ansari and Kumar (2012) reported that soups could be functional enriched foods through replacement of its common ingredients with healthful ingredients; for example, the presence of onion (rich of quercetin) in the formulation of soup samples increases their AA. Substantial amount of phenolic compounds, mainly flavan‐3‐ols as well as flavonoids, in the bread having DKP has been reported (Platat et al., 2015). The intensification of AA caused by the increase in TPC of food products enriched with DKP has been reported before (Amany et al., 2011; Ambigaipalan & Shahidi, 2015; Asghari‐pour et al., 2018; Ghasemi et al., 2020; Platat et al., 2015).

#### 3.3.2 ACE inhibitory activity

Hypertension is an important factor in cardiovascular diseases and due to the important roles of ACE (EC number: 3.4.15.1; Peptidyl‐dipeptidase A) in regulating blood pressure, inhibition of this enzyme is used to treat high blood pressure. ACE inhibitors, similar to synthetic drugs, are widely used to control blood pressure. Blood pressure medications, especially ACE inhibitors, are employed to regulate blood pressure in renin–angiotensin system. Owing to some of the side effects of drug inhibitors in case of long‐term use, natural sources such as plant extracts and bioactive peptides have been considered as alternatives to chemical drugs (Daskaya‐Dikmen et al., 2017).

Table 5 represents the changes in ACE inhibition of soup samples. With the increase in the percentage of DKP, ACE inhibitory activity of soup samples promoted (*p* < .05). ACE inhibitory activities (IC_50_) of S2 and S3 were measured to be 8.2 and 7.86 mg/ml. The addition of DKP to soup samples eventuates minimal ACE inhibition but this activity increases as the percentage of DKP addition intensifies, which could be attributed to the higher polyphenolic compounds and dietary fiber. In fact, phenols act as ACE inhibitors (Iwaniak et al., 2014); Ferulic acid is a weak ACE inhibitor (Al Shukor et al., 2013).

Karakaya, El, Simsek, and Buyukkestelli (2015) confirmed the ACE inhibitory activity in plant product containing seeds and sprouts of lentil, pea, and caseinomacropeptide extracted from whey, and ACE inhibitory activity (37%) of salad dressing containing this plant product was attributed to peptides resulting from hydrolysis of storage proteins by endopeptidases as a result of germination, bioactive peptide glycomacropeptide as well as polyphenol content of dietary plants (Karakaya, El, & Simsek, 2015). Ambigaipalan and Shahidi (2015) reported that the addition of 5% DKP to muffin did not show any ACE inhibition but the level of 2.5% DKP hydrolysate displayed ACE inhibitory activity (app. 13%, IC_50_ = 66 mg/ml); ACE inhibitory activity of the control muffin sample was significantly (app. 36%, IC_50_ = 28 mg/ml) higher than samples containing DKP. Patten et al. (2012) found that ACE inhibitory activity was directly related to TPC and that the processed form of dietary plants showed a wider capacity to regulate the renin–angiotensin system in vitro. Godos et al. (2017) confirmed ACE inhibitory activity by phenolic acids. Hung et al. (2020) reported the highest ACE inhibitory activity in the extracts of different types of bitter watermelon to be 15.8 mg/ml and concluded that with increasing TPC and peptides and triterpenoids in bitter watermelon extract, ACE inhibitory activity intensified. Ambigaipalan et al. (2015) confirmed that protein hydrolysates of date kernel can be used to inhibit ACE activity; IC_50_ values of these hydrolysates were much higher than captopril (a synthetic drug that inhibits ACE).

#### 3.3.3 α‐Amylase and α‐glucosidase inhibitory activities

Slowdown the intestinal absorption of glucose using the inhibition of α‐amylase (EC 3.2.1.1; 1,4‐α‐D‐glucan glucanohydrolase) and α‐glucosidase (EC 3.2.1.20; α‐D‐glucoside glucohydrolase) that decreases the post‐prandial hyperglycemia might represent a noteworthy method for treating type 2 diabetes (Ali Asgar, 2013). The results of inhibition of soup samples against α‐amylase and α‐glucosidase (Table 5) demonstrated a DKP concentration‐depended reduction in α‐ amylase and α‐glucosidase activities (*p* < .05). Samples S2 and S3 showed a significant increase in the inhibition of α‐amylase activity (4.48% and 5.70%, respectively) as compared to control (2.49%). Also, the presence of 2% and 4% DKP improved the inhibition of α‐glucosidase activity in the soups to 4.59% and 6.36%, respectively, in comparison to control (1.06%). So, the highest inhibition appeared in S3, while control showed the weakest effect. The soup samples enriched with DKP exhibited a higher α‐glucosidase inhibitory activity than α‐amylase inhibitory activity. This observation suggests that bioactive compounds inhibiting α‐amylase and α‐glucosidase activity are present in DKP which could be attributed to the polyphenolic compounds and flavonoids. Phenolic compounds have been widely studied as α‐amylase and α‐glucosidase inhibitors (Abdelli et al., 2020; Kang et al., 2014; Kwon et al., 2008; Laoufi et al., 2017; Lou et al., 2018; Meng et al., 2016; Oboh et al., 2016); these compounds interact with digestive enzymes, α‐amylase, and α‐glucosidase (Abdelli et al., 2020); the most cited polyphenolic compounds are quercetin, kaempferol, rosmarinic acid, cyanidin, rutin, catechin, luteolin, and ellagic acid (Egbuna et al., 2021). However, Fratianni et al. (2021) did not find any correlation between the content of total polyphenols and the α‐glycosidase inhibition in honey. Also, the inhibitory effect of flavonoids on key enzymes linked to type 2 diabetes, α‐amylase, and α‐glucosidase has been demonstrated (Kim et al., 2000; Laoufi et al., 2017; Ng et al., 2015; Tadera et al., 2006; Williams, 2013). In general, the weak inhibitory activity of α‐amylase and α‐glucosidase in the soups could be attributed to their low polyphenolic content. α‐amylase and α‐glucosidase inhibitory activities were measured 34.57% and 19.9% for DKP, respectively (Table 1).

### 3.4 Organoleptic properties

Table 6 shows the changes in the scores of organoleptic properties of soup samples. The scores of organoleptic properties (except color) of soup samples increased through increasing the percentage of DKP (*p* < .05). While the highest scores of organoleptic properties (except color) allocated to S3 sample, the highest score of color property belonged to S2 and the lowest score of color property to S3. Date kernel could be used as a food component to improve the sensory properties of food products, and the decrease in water activity affects product stability (Bouaziz et al., 2010). In this research, brightness was reduced as a result of the increase in the percentage of DKP and samples became darker. Texture improvement could be due to higher consistency as a result of the increase in the amount of DKP in soup samples. Food components containing dietary fibers might play a role as texture‐modifying properties in cereal‐based food products, in addition to their functional characteristics. The satisfaction level of panelists of taste might be owing to the interaction of special taste of DKP with other formulation ingredients in soup samples. The soup sample containing 4% of DKP had the highest overall acceptability because of its favorable taste and odor as well as suitable texture. Increased score (of sensory evaluation) and darkness intensity of other products enriched with appropriate amounts of DKP have been reported as well; It should be noted that the color darkness caused by DKP may be desirable or undesirable depending on the product type (Almana & Mahmoud, 1994; Amany et al., 2011; Ambigaipalan & Shahidi, 2015; Ghasemi et al., 2020; Halaby et al., 2014; Shokrollahi & Taghizadeh, 2016; Vinita, 2016).

**TABLE 6:** Organoleptic properties of semi‐prepared dry soup samples* (mean ± standard deviation)

Characteristics treatment	Flavor	Odor	Color	Texture	Overall acceptability
S1 (DKP = 0)	3.7^b^ ± 0.5	3.7^b^ ± 0.5	4.0^b^ ± 0.0	4.0^b^ ± 0.0	3.7^b^ ± 0.5
S2 (DKP = 2)	3.9^b^ ± 0.8	3.9^b^ ± 0.8	4.8^a^ ± 0.4	4.4^a^ ± 0.5	4.0^b^ ± 0.7
S3 (DKP = 4)	4.7^a^ ± 0.5	4.7^a^ ± 0.5	4.1^b^ ± 0.6	4.6^a^ ± 0.5	4.8^a^ ± 0.4

Abbreviation: DKP, Date Kernel Powder (%w/w).

* Means with different subscripts differ significantly (*p* < .05).

## 4 CONCLUSIONS

Dry soup is one of the most popular types of soup due to its longer shelf life and ease of transportation. Currently, the enrichment of foods and beverages with functional natural substances has received much attention from nutritionists, and the use of nutraceuticals in food production brings about human health by reducing the risk of diseases. This study demonstrated that increasing the percentage of DKP in soup samples eventuated the reduction in pH value and promotion of acidity; it resulted in a significant intensification of viscosity while color changes were observed in samples and their fat, fibers, and minerals (iron, zinc, calcium, magnesium, copper, and selenium) raised. With the increase in the percentage of DKP, AA, TPC, and ACE inhibitory activity of soup samples improved. The soup samples exhibited a weak inhibitory activity of digestive enzymes, α‐amylase, and α‐glucosidase.

Finally, the results of this study hint that DKP can be used as a cheap natural source for the production of functional foods such as SPDS based on cereals due to their valuable and functional compounds. For this purpose, the utmost amount of DKP for functional soup production is suggested to be 4% as the color change in products with higher than this threshold amount of DKP is unpleasant; besides, a high level of incorporation causes astringent taste and flavor. It is proposed that dark food products such as meat products, confectionery, chocolates, bakery products, cream‐filled of cakes and biscuits especially biscuits with cocoa and coffee flavors are the best choices for fortification with DKP.

## FUNDING INFORMATION

This research received no specific grant from any funding agency in the public, commercial, or not‐for‐profit sectors.

## CONFLICT OF INTEREST

The authors declare that there is no conflict of interest.

## RESEARCH INVOLVING HUMAN AND ANIMAL PARTICIPANTS

The manuscript does not contain experiments using animals or human studies.

## Data Availability

The authors declare that data supporting the findings of this study are available within the article.
